# Improving gender equity in academia depends on the workplace environment

**DOI:** 10.7554/eLife.105352

**Published:** 2025-04-07

**Authors:** Sreetama Bhadra, Gabriella Damasceno, Daniela Hoss, Alexandra Weyrich

**Affiliations:** 1 German Centre for Integrative Biodiversity Research (iDiv), Halle-Jena-Leipzig Leipzig Germany; 2 Leipzig University Leipzig Germany; 3 Institute of Biology/Geobotany and Botanical Garden, Martin-Luther University Halle-Wittenberg Halle Germany; 4 Evolutionary Genetics, Leibniz Institute for Zoo and Wildlife Research Berlin Germany

**Keywords:** diversity, equity, inclusion, gender bias, careers in academia, research culture, None

## Abstract

The iDiv Female Scientists initiative at the German Centre for Integrative Biodiversity Research (iDiv) Halle-Jena-Leipzig in Germany was set up to connect women in science and to raise awareness of gender inequity. In this article we discuss the impact of the workplace environment on women in academia. Our experiences indicate that supportive workplace environments are more likely to discuss gender inequity, enabling “bottom-up” approaches where individual researchers can propose solutions to the problem. In contrast, unsupportive environments are less receptive to such discussions, so “top-down” approaches driven by legislation policies are required to improve the situation. We also make recommendations of actions that can be taken by individuals, institutions and policymakers to promote gender equity.

## Introduction

Women remain underrepresented in leadership positions in science, technology, engineering, and mathematics (STEM). This is not solely due to unequal recruitment at early career stages (Lerchenmueller and Sorenson, 2018) but also a result of unequal conditions for achieving senior positions, such as professorships (Yildirim and Eslen-Ziya, 2021). Women doing research in STEM fields face gender-related systemic barriers that hinder their professional progress, a phenomenon sometimes called the “leaky pipeline” or “scissors effect” (Grogan, 2018). These barriers include sexual harassment, scarcity of role models, lack of mentoring, neglectful behavior (e.g. being excluded from emails, collaborations and discussions), unequal allocation of working space (offices and laboratories), and the challenges of reconciling working hours with family responsibilities and societal expectations (Ceci et al., 2015; Fouad et al., 2023). These factors create unequal conditions between men and women along their professional careers. To increase the number of women in leadership and decision-making roles in academia, it is essential to implement effective equity measures that address gender differences. Unlike equality, which provides everyone with the same resources and opportunities, achieving equity involves achieving equal outcomes by providing specific resources and opportunities to individuals under different circumstances (Minow, 2021).

In this article, we share insights we gained from leading an initiative called “iDiv Female Scientists (iFS)” at the German Centre for Integrative Biodiversity Research (iDiv) Halle-Jena-Leipzig in Germany. With the iFS initiative, we aimed to raise awareness about the lack of women leaders in academia, strengthen networking among women doing science, provide peer support, and offer workshops to improve technical skills and ultimately support women in their trajectory to leadership positions in academia. Through discussions with over 150 women colleagues at iDiv, diversity committees at three universities (Leipzig University, Friedrich Schiller University Jena, and Martin Luther University Halle-Wittenberg), and senior scientists in other countries, it became clear to us that the role of the workplace environment is often overlooked in discussions of gender equity. We also acknowledge that by focusing exclusively on gender inequity, we overlook the experiences of women who are subject to additional forms of discrimination based on race, nationality and other factors (for more information, see Blithe and Elliott, 2019).

## Influence of the academic workplace environment

The workplace environment for academics includes the physical space where they work (Morgan, 2015), the administrative and technical support available in the workplace, and the institutional values that prevail in the workplace (which may be enshrined in a code of conduct or similar document). This environment can be supportive towards women, or unsupportive, or somewhere in between. Supportive environments are characterized by codes of conduct or similar documents outlining the behavioral norms in the workplace, gender equity training programs, an equal opportunities committee that ensures gender equity in hiring and decision-making processes, career development programs, and support for work-life balance. These elements are absent or only partly present in unsupportive environments (Spoon et al., 2023). Supplementary file 1 includes a proposed (as yet untested) questionnaire that institutions can use to assess how supportive their workplace environment is.

In a supportive workplace environment, employees can adopt a “bottom-up” approach to foster a more inclusive workplace and promote gender balance in leadership roles. This approach can start with acknowledgement, followed by transparency and action. In contrast, unsupportive workplace environments tend to perpetuate the *status quo* of men in leadership positions by offering limited support to women. In such unsupportive environments, a “top-down” approach that starts with policymakers (such as government departments and funders) or whole institutions taking action is more likely to lead to change. Table 1 lists a range of specific steps in the areas of acknowledgement, transparency and action that can be undertaken by individual researchers, by universities and research institutions, and by policymakers.

**Table 1.** Guidelines of specific steps to achieve acknowledgement, transparency and action. At the level of individual researchers, the points in blue text indicate guidelines we performed and experienced within the iFS initiative; at the level of universities and research institutions, the points in blue text indicate topics in which the involvement of iFS resulted in improvements in equity at our institute; and at the level of policymakers, the points in blue text are topics we expect this paper can have an impact on. The points in black text are based on discussions we had with other women in science within iFS, on perspectives we see for the future, and on ideas we found in literature (such as Grogan, 2018 and Snickare et al., 2022).

Individual researchers		
Acknowledgement	Transparency	Action
**Recognize the problem of gender inequity and become informed on how to tackle it:** Learn about the leaky-pipeline and scissors effect problems Familiarize yourself with local initiatives addressing the problems, like equity committees	**Increase collective awareness:** Foster discussions and workshops about biases and gender inequity Spread information about local initiatives	**Support women:** Nominate and vote women for leadership positions Foster inclusivity criteria in hiring processes
**Become aware of your own biases**: Attend unconscious bias workshops Quantify gender bias among authors and author roles in your own manuscripts	**Be open about your own biases:** Critically reflect on your own biases Discuss biases with colleagues	**Explicitly address biases:** Invite more women peers to collaborate Suggest/ create a code of conduct” for your own group
**Be attentive to your surroundings:** Calculate the proportion of women in the institution, especially in leadership positions Become informed about regulations to handle discrimination and harassment complaints	**Promote a friendlier environment:** Adopt an active by-stander behavior Demand transparency in handling discrimination/ harassment complaints	**Ensure a safer environment:** Adopt non-discriminatory language and practices Intervene or report cases of discrimination/ harassment
Universities and research institutions		
Acknowledgement	Transparency	Action
**Collect and acknowledge gender-disaggregated data:** About infrastructure, and financial and human resources available to employees On staff productivity (funding, publication, career stage, career progression) in relation to the resources available to them	**Increase awareness:** Make gender-disaggregated data available for staff on intranet or internet portals Implement mandatory workshops about unconscious bias for employees	**Reduce inequity:** Implement inclusive criteria for hiring women and adopt alternative metrics to evaluate their performance (Re-)distribute resources with equity as the guiding criteria
**Collect data on discrimination and harassment complaints**: Create periodic anonymous surveys for collecting data about discrimination/ harassment Standardize the way data about discrimination/ harassment is collected at different sources, like departments and research groups	**Share the data safely:** Inform staff about the due process for dealing with discrimination/harassment complaints Report the outcomes of the due process (i.e., number of complaints, type of outcome) without disclosing identities of victims	**Implement measures against discrimination/ harassment:** Establish a system of “ checks and balances ” to prevent power structures from interfering with the due process dealing with discrimination/ harassment Implement a code of conduct establishing a clear and time-bound process in cases of discrimination/ harassment; preferably written together with people who are vulnerable to misconduct
**Assess policies and plans towards equity:** Critically evaluate what has been implemented and achieved Gain inspiration from successful initiatives	**Plan together with the community to achieve gender equity:** Set up and publicize action plans with clear goals and milestones Specify a timeframe for evaluation and revisions of plans	**Guarantee implementation of plans:** Devote human and financial resources to implement and review the plans periodically Reward departments who achieve the goals, and demand adaptation/ explanation from those who do not
Policymakers		
Acknowledgement	Transparency	Action
**Gather and harmonize data about the problem:** Compile and harmonize gender-disaggregated data at the federal and national level, leveraging the data already collected by institutions and international organizations Identify institutions and research fields in which actions towards equity are required	**Create standards for data collection at institutions:** Create guidelines for data collection and report, with standardized terminology of career positions Demand annual reports from institutions containing gender-disaggregated data	**Set actions and goals to be taken by institutions:** Demand institutions to create plans and policies for gender equity Mandate data sharing by institutions
**Collect information about existing public policies targeted to gender inequity:** Critically assess existing policies, including evaluation and recommendations by experts Assess how institutions and countries are performing in comparison to international guidelines for gender equity	**Create an agenda of inclusivity:** Create plans for the development and implementation of regulations to deal with gender inequity Establish a “reputation rank” for universities and institutions, ranking them regarding their support for women and equity initiatives	**Establish regulations and legal frameworks to foster implementation of gender equity in science:** Create legal security for women in science, for example, paid maternity leave Create support initiatives targeted to keep women in science, for example providing child-care spaces and allowing working flexibility to accommodate family-care duties
**Inspect and review funding grants in relation to gender:** Gather gender disaggregated data on grant awardees, preferably by research fields and institutions Inspect for biases in grant awards, including the type and amount of grants	**Promote transparency in funding:** Publicize the number of grant applicants/nominees and awardees by gender and knowledge field Disclose criteria used for grant awards, especially those preventing/ promoting gender equity	**Create system of rewards and penalties for institutions:** Constrain awarding of grants to the success of institutions to implement gender equity measures Create awards for gender equity initiatives

## Acknowledgement, transparency and action

**Acknowledgement** of our conscious and unconscious biases, and recognition of the problem of gender inequity, are crucial steps when seeking to drive change. At the individual level, we can make unconscious assumptions about people based on various characteristics (Fiarman, 2016). For example, biases often lead to the unfounded perception that women are less intelligent and capable than men (Furnham and Rawles, 1995). This is evidenced in the Nobel Prizes for science and medicine, in which men are more commonly nominated and awarded (Tol, 2024). Since conscious and unconscious biases influence decision-making (Oeberst and Imhoff, 2023), individual biases can contribute to the underrepresentation of women in leadership positions. Acknowledgement of these facts is necessary to increase awareness and pave the way for meaningful change. Individuals can, for example, recognize gender inequity as a real issue, become informed on ways to address it, be aware of their own biases, and be attentive to their environment. Universities and research institutions can collect and analyse data on gender disparities, track reports of discrimination and harassment, and assess the effectiveness of policies and strategies aimed at achieving equity.

**Transparency** is a core step between acknowledging biases and implementing actions. To achieve transparency, individuals need to disclose biases and support an inclusive environment, thus contributing to collective awareness. Universities and research institutions can promote transparency by sharing data and collaborating with their communities to achieve gender equity. Policymakers can establish standardized guidelines for data collection across institutions, develop inclusion agendas, and promote transparency in funding (Table 1). When data on gender disparities are being collected, analyzed and reported, it is important to take career stages into account because gender parity is common among students and for those in relatively junior positions, but substantial disparities arise at more senior levels (see Lu et al., 2021; Supplementary file 2).

In a bottom-up approach, **actions** should rely on transparent data and foster collaboration among researchers, institutions and policymakers. At an individual level, actions include supporting women in their career progress and explicitly addressing biases. Universities and research institutions can reduce inequity by enforcing measures against discrimination or harassment, ensuring implementation of equity plans and guaranteeing a safer workplace environment. Policymakers can set clear goals and actions for institutions, establish regulations and legal frameworks for implementing gender equity in academia, and create systems of rewards and penalties applied to institutions to encourage their compliance (Table 1).

## The role of supporters

Supporters are essential in advancing gender equity and promoting women in leadership positions. Their proactive involvement, both within the institutional system and in the broader community, can challenge discriminatory norms and foster a more inclusive environment. These supporters are individuals and organizations who actively engage in and act to support a cause. We recognize the following supporters:

**Men**: Men frequently hold decision-making positions in academia, granting them influence over resources (Farre, 2013). To promote gender equity, men can, for example, decline to join men-only panels, support women who face individual or systemic gender discrimination and sexual harassment, and amplify women’s voices when they bring attention to challenges and problems (Wade and Zaringhalam, 2019).

**Scientific societies**: Discipline-focused scientific societies are well-placed to raise awareness of and address gender disparities because they bring together institutions, countries, cultures, and individuals. We recommend that societies have policies and structures that address gender-related problems, such as the underrepresentation of women, discrimination and sexual harassment. Societies can also contribute by transparently reporting gender representation in leadership positions within the society, principal investigators funded by the society, and institutional workplace environment rankings. Additionally, societies should offer anonymous reporting services for discrimination and sexual harassment, supported by a clear action plan with a time-bound resolution process. To reinforce women’s representation at scientific conferences, societies can also: (i) invite equal numbers of men and women to be keynote and plenary speakers at meetings (Shishkova et al., 2017); (ii) address the problems that prevent women from attending meetings, such as a lack of child-care facilities; (iii) establish a safe, confidential space for reporting sexual misconduct with prompt and time-bound actions guided by a code of conduct. Finally, societies should explicitly consider applicants' commitment to gender equity actions in their evaluations for funding and awards.

**Scientific journals and popular science magazines**: When commissioning articles, scientific journals should invite equal numbers of men and women to write and review articles, as well as adopting double-blinded peer-review (Budden et al., 2008). Popular science magazines can also promote transparency by publishing articles that contain data on gender disparities, or articles that rank institutions and countries based on how supportive their environments are (Djerf-Pierre, 2011). Additionally, publishing anonymous reports on misconduct in institutions can reduce discrimination against those who report it because evidence shows that women reporting sexual misconduct and discrimination face greater professional penalties (Tollefson, 2023) compared to men who commit such offenses (Balter, 2023).

**Social media**: Social media enables isolated women to connect with global networks of like-minded individuals, turning their experience into opportunities for idea-sharing and support. Initiatives such as 500 Women Scientists (https://500womenscientists.org/) and social media groups like STEMPeers (https://stempeers.org/) offer platforms for discussing individual and systematic problems, with options for anonymous participation. It might also be possible for websites aimed at academics (such as ResearchGate), to team up with employment websites (such as Glassdoor), so that current and former employees can anonymously share insights about academic institutions.

## Conclusion

Women remain underrepresented in leadership positions in STEM fields within academia, despite their increasing participation in recent years. Unsupportive workplace environments force many women to leave academia as they advance in their careers. Acknowledging gender inequity – whether as individuals, institutions, or policymakers – is crucial for addressing the issue. Transparency reveals the scale of the problem, and the commitment to act can lead to meaningful change. Individual networking initiatives, such as ours, can spotlight gender inequity, but these efforts are most effective in supportive environments – although there is still a long way to go.

## Data Availability

There are no data associated with this article.

## Additional files

Supplementary file 1. Questionnaire for evaluating the supportiveness of workplace environments.

Supplementary file 2. Collection of gender-disaggregated data.
