# Supplementary material for: Improving gender equity in academia depends on the workplace environment
Source: eLife. 2025 Apr 7;14:e105352. doi: 10.7554/eLife.105352 (PMC11975367; doi:10.7554/eLife.105352)
Supplement: Supplementary file 1. [file elife-105352-supp1.docx]

Bhadra S, Damasceno G, Hoss D, Weyrich A. 2025. Improving gender equity in academia depends on the workplace environment *eLife* **14**:e105352 DOI: 10.7554/eLife.105352

# **Supplementary file 1**

# **Questionnaire for evaluating supportiveness of workplace environment**

# A suggestive questionnaire to evaluate the supportiveness of the workplace environment. In this format, workplace environments can fall within a continuum of supportive (47 points), neutral (0 points), or unsupportive environments (-50 points). The questionnaire can be used by a single person (or a group of people) to assess their workplace environment or by institutions to collect institutional-level data. In this case, institutions can adapt the questionnaire to their specific realities and needs.

### **Section 1: Demographics and Background Information**

# Age:

# Under 25

# 25-34

# 35-44

# 45-54

# 55+

# Gender:

# Man

# Woman

# Non-binary

# I prefer not to say

# Current Position:

# Undergraduate Student

# Graduate Student

# Postdoc

# Faculty

# Administrator

# Other (please specify)

### **Section 2: Institutional Code of Conduct**

# Is there a clear Code of Conduct in your institution?

# Yes (1)

# No (-1)

# Not sure (0)

# Are there clear, formal gender-equity policies in place at your institution?

# Yes (1)

# No (-1)

# Not sure (0)

# Does your institution offer gender-equity training programs (e.g. unconscious bias and bystander workshops)?

# Yes (1)

# No (-1)

# Not sure (0)

# Does your institution have and adopted measures to promote gender equity in its hiring practices?

# It has and I know they are adopted measures (2)

# It has but I do not know if the measures are adopted (1)

# It has but I think the measures are not adopted (-1)

# It doesn’t have any measure (-2)

# I don’t know (0)

# Are there mentorship programs aimed at supporting gender equity at your institution (e.g. leadership programs for women in science)?

# Yes (1)

# No (-1)

# Not sure (0)

# Do you have access to mentorship or professional development programs that support your career growth?

# Yes (2)

# Somewhat (1)

# No (-1)

- - Not sure (0)

# Do you feel that men and women are equally supported in advancing to leadership positions in your organisation?

# Yes, definitely (2)

# Somewhat (1)

# No (-1)

# Unsure (0)

# Do the leaders at your institution promote and support gender equity initiatives?

# Yes, consistently (2)

# Occasionally (1)

# No (-1)

# I’m not aware (0)

# How do you judge the effectiveness of gender-equity policies at your institution?

# Very effective (2)

# Somewhat effective (1)

# Ineffective (-1)

# Very ineffective (-2)

# Neutral (0)

### **Section 3: Personal Experiences**

# Have you ever experienced gender-based discrimination at your institution?

# Yes (-1)

# No (1)

# Prefer not to say (0)

# If yes, in what context(s)? (Select all that apply. Every option counts as -1 point)

# Recruitment and hiring

# Promotion and career progression

# Salary and bonus

# Mentorship opportunities

- - Exclusion from authorship
  - Exclusion from cooperation

# Work-life balance

# Leadership opportunities

# Other (please specify)

# How would you rate the level of support provided to women and gender minorities at your institution?

# Very supportive (2)

# Somewhat supportive (1)

# Somewhat unsupportive (-1)

# Very unsupportive (-2)

# Neutral (0)

# Do you feel your voice is heard in decision-making processes related to gender equity?

# Yes, always (2)

# Sometimes (1)

# Rarely (-1)

# Never (-2)

- - I am not sure (0)

# Do you feel that colleagues that are men are treated more favourably than women or gender-minority colleagues at your institution?

# Strongly agree (-2)

# Agree (-1)

# Disagree (1)

# Strongly disagree (2)

# Neutral (0)

1. Is there a clear, accessible process for addressing gender-based concerns or grievances in your workplace?
   - Yes (2)
   - Somewhat (1)
   - No (-1)
   - I’m not sure (0)

### **Section 4: Workplace Culture**

# Do you feel that your workplace encourages a culture of inclusivity and diversity?

# Strongly agree (2)

# Agree (1)

# Disagree (-1)

# Strongly disagree (-2)

# Neutral (0)

# Do you feel respected by your colleagues regardless of your gender, background, or role?

# Yes, always (2)

# Sometimes (1)

# Rarely (-1)

# Never (-2)

- - I am not sure (0)

# 3. Is there open communication and collaboration among employees at all levels?

# Strongly agree (2)

# Agree (1)

# Disagree (-1)

# Strongly disagree (-2)

# Neutral (0)

# 4. How often do you witness or hear about gender biases in everyday interactions at your institution?

# Frequently (-2)

# Sometimes (-1)

# Rarely (1)

# Never (2)

- - I don’t know (0)

# 4. Do you believe that your institution is committed to addressing gender inequity?

# Yes, very committed (2)

# Somewhat committed (1)

# Not committed (-1)

# Neutral (0)

# 5. How would you describe the overall atmosphere regarding gender issues at your institution?

# Very positive (2)

# Somewhat positive (1)

# Somewhat negative (-1)

# Very negative (-2)

# Neutral (0)

### **Section 5: Supportive vs Non-Supportive Environments**

# Do you feel your institution provides adequate support for work-life balance, particularly for women and gender minorities?

# Strongly agree (2)

# Agree (1)

# Disagree (-1)

# Strongly disagree (-2)

# Neutral (0)

# How accessible are leadership opportunities for women and gender minorities at your institution?

# Very accessible (2)

# Somewhat accessible (1)

# Somewhat inaccessible (-1)

# Very inaccessible (-2)

# Neutral (0)

# Do you believe supportive environments contribute positively to the retention of women and gender minorities in your institution?

# Strongly agree (2)

# Agree (1)

# Disagree (-1)

# Strongly disagree (-2)

# Neutral (0)

# In your experience, how do non-supportive environments impact career progression for women and gender minorities?

# Severely hinder (2)

# Somewhat hinder (1)

# Do not hinder (-1)

# Neutral (0)

# Are there policies in place that support family leave, flexible hours, or other accommodations for balancing work and personal responsibilities?

# Yes, and they are accessible (2)

# Yes, and they are somewhat accessible (1)

# Yes, but they are difficult to access (-1)

# No (-2)

# I’m not aware (0)

# In comparison to other institutions, how would you rate your current institution’s efforts to foster a supportive environment for gender equity?

# Much better (2)

# Better (1)

# Worse (-1)

# Much worse (-2)

# About the same (0)

### **Section 6: Suggestions and Feedback** (To be used by institutions when collecting data)

# What changes would you recommend improving gender equity at your institution?

# *(Open ended)*

# What specific support mechanisms do you believe are necessary for creating a gender-equitable environment in scientific institutions?

# *(Open ended)*

# Please share any additional comments or experiences related to gender equity in your institution.

# *(Open ended)*
