# Supplementary material for: Improving gender equity in academia depends on the workplace environment
Source: eLife. 2025 Apr 7;14:e105352. doi: 10.7554/eLife.105352 (PMC11975367; doi:10.7554/eLife.105352)
Supplement: Supplementary file 2. [file elife-105352-supp2.docx]

Bhadra S, Damasceno G, Hoss D, Weyrich A. 2025. Improving gender equity in academia depends on the workplace environment *eLife* **14**:e105352 DOI: 10.7554/eLife.105352

**Supplementary file 2**

**Collection of gender-disaggregated data**

Academia is structured hierarchically, with few permanent positions that hold decision-making power – most of which are held by men. Therefore, when studying gender bias, it is crucial to collect, analyse and report gender disaggregated data. Gender aggregated data often creates a misleading impression of gender and power balance, as shown in the gender-aggregated data (left panel) and a conventional categorization (lower right panel). Our example shows equal numbers of women and men working in a scientific institute (150 each; left panel). When disaggregating the data by role (upper right panel), it can be seen that women outnumber men in junior positions such as PhD students, that there is equal representation among postdoctoral researchers, whereas men hold most senior positions (e.g., senior scientists). Additionally, power structures can be further obscured by status-related roles such as principal investigator (PI), co-PI, or collaborator. Here, we propose the suggested categorization (upper right panel) to ensure a clearer understanding of gender distributions. We recommend using a uniform categorization system that distinguishes between researchers and supporting scientific staff (such as administrative staff), career stage (PhD, postdoctoral researchers, senior scientists), as well as between permanent and temporary positions, reflecting employment security. This categorization will help reveal how gender disparities manifest at different levels of the academic hierarchy, particularly within institutions.
